# Modern treatment in chronic lymphocytic leukemia: impact on survival and efficacy in high-risk subgroups

**DOI:** 10.1002/cam4.226

**Published:** 2014-03-19

**Authors:** Antonio Cuneo, Francesco Cavazzini, Maria Ciccone, Giulia Daghia, Olga Sofritti, Elena Saccenti, Massimo Negrini, Gian Matteo Rigolin

**Affiliations:** 1 Hematology Section, Department of Medical Sciences, University of Ferrara, University Hospital Arcispedale S. AnnaFerrara, Italy; 2 Laboratory for Technologies of Advanced Therapies (LTTA) and Department of Morphology, Surgery and Experimental Medicine, University of FerraraFerrara, Italy

**Keywords:** Chronic lymphocytic leukemia, chemoimmunotherapy, genetic lesions, tyrosine-targeted treatment, BCL2

## Abstract

Treatment of chronic lymphocytic leukemia (CLL) has dramatically changed over the last years, with significant improvement in overall survival (OS) and increased efficacy in genetically defined “high-risk” disease. Besides prospective clinical trials usually enrolling young and fit patients, retrospective studies were performed comparing the outcome of patients belonging to different age groups and showing longer survival in patients diagnosed in the most recent periods. In patients younger than 70 years the 10-year relative survival was 43–53% in the 1980s as compared with 59–63% in the 2000s. Likewise, the 10-year relative survival in patients >70 years was 22–42% in the 1980s and 46–55% in the 2000s. Improved outcome derived in part by the introduction of effective regimens in genetically defined “high-risk” disease (i.e., 17p−, 11q−, *TP53*, *NOTCH1*, *SF3B1* mutations), especially in the younger and/or fit patients. The unfavorable prognostic significance of 11q− was overcome by chemoimmunotherapy. High-dose steroids with anti-CD52 appeared to improve the response rate in 17p-/*TP53* mutated cases and allogeneic transplantation achieved prolonged disease control irrespective of high-risk disease. Further improvement is being generated by the new anti-CD20 obinutuzumab in the elderly and by mechanism-based treatment using kinase-targeting agents or anti-BCL2 molecules yielding high-response rate and impressive progression-free survival in the chemorefractory setting as well as in previously untreated patients.

## Introduction

Treatment of chronic lymphocytic leukemia (CLL) has dramatically changed in several respects over the last years thanks to the convergence of basic research and well-conducted clinical studies leading to a clearer understanding of pathophysiology of the disease, to the identification of prognostic factors and to the design of effective treatment regimens 1–12.

Modern regimens produced high overall response rates (ORR), including complete remissions with negativity for minimal residual disease (MRD) and prolonged progression-free survival (PFS). The combination of rituximab fludarabine and cyclophosphamide (FCR) was shown to be superior to FC for all clinical endpoints including overall survival (OS), with the notable exception of the 17p− and the “normal FISH” subgoups 13. Meanwhile, evidence was provided that some disease subsets defined by molecular cytogenetic lesions represent “high-risk” disease with shorter PFS and survival with current treatment regimens 14–17. Novel agents interfering with unique biologic features, that is, B cell receptor (BCR) downstream signaling and *BCL2*, are being rapidly introduced in clinical practice, representing a new scenario of mechanism-driven treatment of CLL, producing rapid and durable responses in relapsed/refractory CLL 18,19.

With some exceptions 20, most clinical studies enrolled relatively young and fit patients and to address the issue of whether modern treatment produced a survival benefit in all age groups several retrospective studies were performed 21–23.

Modern treatment approaches will be reviewed here with reference to

1. their impact on OS in different age groups and
2. their activity in specific molecular cytogenetic subsets.

## Impact of Treatment on Survival

Survival of the general population improved in the last decades in many western countries 24 and several factors may influence survival in historical series, including earlier diagnosis due to widespread use of automatic blood counters, more precise diagnosis allowing for the exclusion of lymphoma in leukemic phase in recent years, and improved supportive treatment. However, the following observations indicate that the overall outlook of CLL improved in the majority of age groups over the last decades.

### Data from single centers and registries

**Figure 1:**
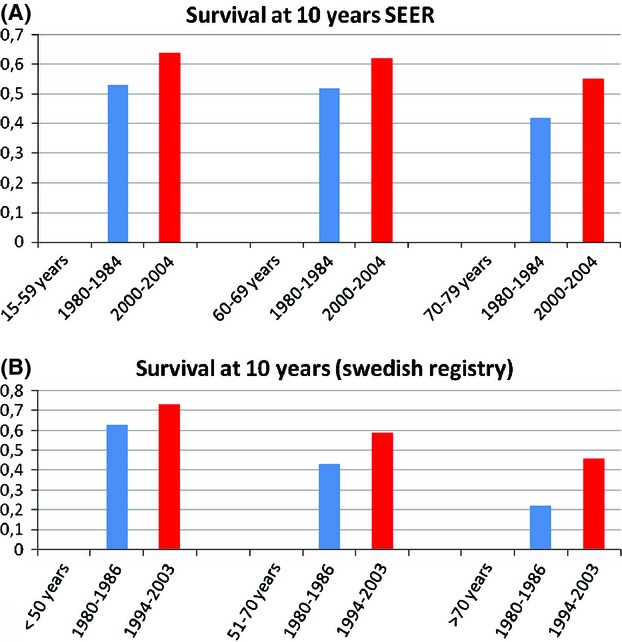
Improved 10-year survival in different age groups. Survival is expressed as ratio of absolute survival of CLL patients divided by the expected survival of a comparable group of persons in the general population. Data from (A) Brenner et al. 21 and (B) the Swedish registry 23. CLL, chronic lymphocytic leukemia.

Brenner and coworkers 21 assessed relative survival rates in CLL calculating the ratio of absolute survival of CLL patients divided by the expected survival of a group of well-matched persons in the general population. An improvement in survival in patients <80 years between 1980–1984 and 2000–2004 was documented in this analysis (Fig. 1A). In 2000–2004, patients <70 years reached a 10-year relative survival close to 65%, whereas a 55% 10-year relative survival was reached in the 70–79 age group.

The CLL-attributable mortality for patients diagnosed in 1995–2004 and 1980–1994 was calculated in the Barcelona series as deaths per 1000 patient-year, with an incidence rate ratio of 0.46 and 0.65 at 5-years and 10 years, respectively 22. Improved 5- and 10-year relative survival in the 1995–2004 period as compared with the 1980–1994 period was more pronounced in stage B/C patients <70 years. These data suggest that more effective treatment produced longer survival in young patients with intermediate-advanced stage, whereas no obvious improvement was noted in this analysis in limited-stage disease and in the elderly.

Using population-based data in an efficient Swedish registry, Kristinsson and coworkers 23 assessed variations in survival among CLL patients and found significantly improved 5-, 10-year relative survival ratio for the entire cohort during the study period for the majority of the age groups (Fig. 1B). Stable age-adjusted incidence and stable mean age at CLL diagnosis over the study period seem to outrule lead time bias due to early CLL detection as confounding factor in this analysis; however, improved survival in the general population, including CLL patients, might have played a role here. An unexplained observation in this study was that the 5-year relative survival ratio improved only in the 1973–1980 period and was stable thereafter in the youngest CLL population. Interestingly, adverse prognostic markers were found to be more common among young patients 25 and this might have influenced the outcome in this age subset in an era preceding the widespread use of biologic agents.

### Comparisons with historical controls

Chemoimmunotherapy upfront was found to prolong survival at 6 years (77%) with respect to previous trials using fludarabine-based treatment (54–59%) 12. More recently, PFS and OS were retrospectively assessed in four successive frontline CALGB trials 26. With a median follow-up across studies of 92 months, OS was improved with fludarabine over chlorambucil (31% reduction of risk of death) among patients <70 years, but not in older adults. Importantly, a 35% reduction of risk of death was observed with the adjunct of rituximab to fludarabine, irrespective of age.

### Randomized trials

At an extended follow-up analysis with a median observation of 5.9 years in the CLL8 trial, 69.4% of the patients were alive in the FCR group versus 62.3% in the FC group 27. Inclusion criteria in this protocol precluded enrollment of many elderly patients and when restricting outcome analysis in the 30% study population ≥65 years, improved complete remission (CR) rate and PFS were maintained in the chemoimmunotherapy arm, whereas no significant advantage in survival was noted in this age subset.

Although no difference in survival was observed in a trial comparing fludarabine versus chlorambucil in the elderly 28, a planned interim analysis of the CLL11 trial designed for unfit patients and comparing chlorambucil versus chlorambucil plus rituximab or the novel anti-CD20 monoclonal antibody obinutuzumab, found survival advantage in the chlorambucil plus obinutuzumab arm as compared with chlorambucil 20. Notably improved PFS was recorded in the obinutuzumab arm as compared with the rituximab 20. These data overall indicate that true improvement in survival is nowadays achievable in the majority of age groups, especially for those patients eligible to chemoimmunotherapy.

## Efficacy of Treatment in Specific Molecular Cytogenetic Subsets of CLL

There is evidence deriving from single-center studies and from prospective multicenter trials that specific molecular-cytogenetic lesions, that is, 17p− 11q−, *TP53*, *NOTCH1*, and *SF3B1* mutations occur in all age groups and may predict for chemorefractoriness and worse prognosis 29–36. Improved outcome in CLL derived in part by the introduction of novel regimens which proved to be effective in all risk categories, including genetically defined “high-risk” disease (i.e., 17p−, 11q−, *TP53*, *NOTCH1*, *SF3B1* mutations). These regimens were tested preferentially in younger and/or fit patients. Efficacy data of chemoimmunotherapy in the frontline setting in distinct cytogenetic subsets are presented in Table 1.

**Table 1:** Efficacy of the main frontline treatment regimens in different cytogenetic subsets of CLL

Reference	Regimen	Response rate expressed as %ORR/%CR					Survival expressed as PFS/OS (months)				
	
		All groups	13q−	+12	11q−	17p−	All groups	13q−	+12	11q−	17p−
Hillmen et al. 80	Chlorambucil	55.4/NR	62/NR	80/NR	29/NR	20/NR	11.7/NR	13/NR	12.9/NR	8.5/NR	2.2/NR
Hillmen et al. 80	Alemtuzumab	83.2/NR	91/NR	83/NR	87/NR	64/NR	14.6/NR	24.4/NR	18.3/NR	8.5/NR	10.7/NR
Hallek et al. 13	Fludarabine cyclophosphamide	80/22	80/23	84/19	87/15	34/0	45%/83%*	52%/89%*	48%/86%*	32%/83%*	0%/37% *
Hallek et al. 13	Fludarabine cyclophosphamide rituximab	90/44	96/48	100/71	93/51	68/5	65%/87% *	76%/95%*	83%/96%*	64%/94%*	18%/38%*
Bosch et al. 81	Rituximab fludarabine cyclophosphamide mitoxantrone	82/11	NR/82	NR/100	NR/87	NR/25	NR	NR	NR	NR	NR
Parikh et al. 82	Cyclophosphamide fludarabine alemtuzumab rituximab	92/70	100/64	100/93	90/80	78/57	38/NR	42**/NR	42**/NR	27/NR	15/NR
Fisher et al. 69	Rituximab bendamustine	88/23	93.3/13.3	94.7/21	90/40	37.5/0	33.8/NR	34.4/NR	Not reached/NR	29.7/NR	7.9/NR
Pettitt et al. 39	Alemtuzumab methylprednisolone	NA	NR	NR	NR	88/65	NA	NR	NR	NR	18.3/39

CLL, chronic lymphocytic leukemia; ORR, overall response rate; CR, complete remission; PFS, progression-free survival; OS, overall survival; NR, not reported; NA, not applicable.

* At 3 years;

** median not reached.

Over the last few years, however, the introduction of novel treatment regimens and the recent development of molecules interfering with specific biologic mechanisms changed the treatment paradigm in CLL 37, especially in high-risk disease and chemorefractory disease. The efficacy and safety of novel regimens in the relapsed-refractory setting including the “unfavorable” genetic subsets of CLL is illustrated below and summarized in Table 2.

**Table 2:** Efficacy and safety of some classical and novel treatment options in relapsed refractory CLL

	Regimen (reference)										
	
	Various regimens 83	FCR 84,85	Ofatumumab 86	Lenalidomide + R 62	Ibrutinib 9	Idelalisib + R/B/RB 51	ABT-199 57	B + R 72		R-BAC 41	Flavopiridol 42
Number of patients	99	276/284	138	59	85	51	56	78		13	40
Number previous regimens (median)	NA (fludarabine refractory)	1/2	4 (fludarabine refractory)	2	4	1–10 (range)	4	2		3	4
Response											
CR	0%	24/30%	0–1%	12%	2%	78–87% (ORR)	21%	9%		38%	46% (ORR)
PR	23%	45/44%	47–58%	54%	69%(iii)		63%	50%		46%	
Follow-up (i)	NA	25/43	NA	33	26	>40 weeks	NA	24		17	NA
PFS	2–3	30/21	5.7–5.9	17,4 (ii)	75%(iv)	74–87%(iv)		15.2		16	10.4
Survival	9	NR/47	13.7–15.4	71%	83%	NA		33.9		NR	19.8
Grade 3/4											
infections	54%	18/16%	8–12%	24%	17%	0–29% (v)	7% (vi)	0–3.4%		8%(vi)	NA
neutropenia	NA	89/81%	6–14%	73%	15%	32–67%	41%	4.8–5.4%		84%	NA

PFS, progression-free survival; CLL, chronic lymphocytic leukemia; NA, not available; NR, not reached; ORR, overall response rate; R, rituximab; B, bendamustine; (i) months, median value; (ii) time to treatment failure; (iii) an additional 18% patients had PR with lymphocytosis; (iv) % at 26 months for ibrurinib and % at 1 year for idelalisib; (v) pneumonia; (vi) febrile neutropenia.

## 17p−/*TP53* Mutations

This subset of CLL is mostly refractory to fludarabine and alkylating agents and shows, with few exceptions 38, a poor prognosis with expected median survival of few years even with intensive regimens. Because the anti-CD52 monoclonal antibody alemtuzumab and high-dose steroids kill CLL cells through a p53 independent mechanism the efficacy of these drugs in combination was assessed 39, producing a 65% CR rate, with 36% MRD-disease and PFS median of 18.3 months in untreated patients. Despite representing a progress with respect to other regimens, virtually all patients are expected to relapse.

Allogeneic transplantation is an option for these patients. Interestingly, 6-year OS and event-free survival were 58% and 38%, respectively, in a study of 90 allografted high-risk patients, 49% of whom were fludarabine resistant. The efficacy results of this procedure were independent of the presence of unfavorable genetic features, including 17p− 40.

The combination of rituximab, bendamustine, and cytarabine in nine heavily pretreated patients with 17p− achieved CR in three cases and PR in four, with an ORR of 78% and a median PFS of 16 months in the entire series including four additional patients with 11q− 41. Flavopiridol as single agent attained a 48% ORR in 40 pretreated patients with 17p− with median PFS of 10.4 months; these data were not significantly different among the cytogenetic groups included in the study 42.

Novel agents showed promising efficacy in this cytogenetic subsets of CLL as summarized below.

### BCR-Targeted Therapy

#### Ibrutinib

The Bruton tyrosine kinase (BTK) is a cytoplasmic tyrosine kinase that is essential for BCR signaling, inducing cell proliferation, and activation of the NF-κB pathway. Ibrutinib is an oral agent which binds covalently to Cys-481 of BTK, causing its inhibition.

The publication by Byrd and coworkers 9 of a phase Ib-2 multicenter study to assess the safety and efficacy of ibrutinib in 85 relapsed-refractory CLL who had received a median of four previous lines of treatment was welcomed as the first mechanism-driven treatment for CLL 18.

The drug induced rapid shrinkage of lymph nodes with increase in the absolute lymphocyte count, reflecting a compartment shift. Over time, this lymphocytosis gradually resolved in the majority of the cases.

Toxicity was modest (Table 2), with grade 1–2 diarrhea, fatigue, and upper respiratory tract infection being the most common events.

Responses were independent of stage, number of previous therapies, and 17p−. At 26 months an impressive 75% PFS and 83% OS were observed. In this and in another phase II trial 43, there was no apparent difference in the incidence of response between patients with and without 17p−. However, disease progression occurred in 11 patients in the trial by Byrd and coworkers 9, 10 of whom had 17p− or 11q−. Interestingly, whole exome sequencing at baseline and after disease progression showed single nucleotide variations in three patients in the relapse sample 44. Two patients had distinct mutations that encoded a cysteine-to-serine substitution at position 481 of BTK (C481S) and the third patient acquired a potential gain-of-function mutation encoding a R665W substitution in PLCg2, a substrate of BTK, consistent with constitutive PLCg2 activation. Although rare, the acquisition of C481S BTK and R665W PLCg2 mutations in the setting of resistance suggests mechanisms of ibrutinib resistance. In another study 45, resistance to ibrutinib was observed in patients showing clonal evolution with the appearance of driver SF3B1 mutations or 8p deletion arising from a background of preexisting 17p− or 11q−.

The favorable therapeutic index, along with its tolerability and efficacy in the first-line setting 46 may facilitate the use of ibrutinib in combination with other agents to limit the increase in peripheral lymphocytosis and to further improve its efficacy 47,48.

#### Idelalisib (GS1101–CAL101)

CAL-101 inhibits PI3K-Δ, causing apoptosis in CLL cells, sparing T-cells or NK cells. In vitro, CAL-101 was able to sensitize CLL cells to the effects of cytotoxic drugs and steroids and to interact with BCR signaling, possibly reflecting a dual mechanism of action 49.

A clear benefit of idelalisib and rituximab over rituximab alone was documented independent of the presence or absence of 17p− in heavily pretreated patients who were not able to receive chemotherapy due to cytopenias or comorbidities 50. Furthermore, Coutre and coworkers 51 demonstrated durable responses in the majority of patients using idelalisib in combination with rituximab and/or bendamustine. As with ibrutinib, nodal response was associated with lymphocytosis; this effect was limited by adding ofatumumab in one study of 15 patients producing a 94% ORR 52. The favorable safety profile of idelalisib allowed the administration of this oral PI3K-Δ inhibitor at the full single dosage with concomitant chemoimmunotherapy and provided the basis for the initiation of studies evaluating its efficacy in combination with rituximab or bendamustine ± rituximab, with fludarabine or chlorambucil. Idelalisib showed robust activity independent of the presence of 17p− both in pretreated and in untreated patients 53,54.

#### BCL2 Antagonists

*BCL2* is overexpressed by CLL and plays an antiapoptotic role 55. The *BCL2* gene antisense nucleotide oblimersen did not produce significant survival advantage in an intent to treat analysis 56. More recently, the Bcl-2 antagonist ABT-263 showed activity in CLL with dose-limiting thrombocytopenia due to concomitant Bcl-xL inhibition. A single dose of the compound ABT-199 targeting more specifically Bcl-2 resulted in potent tumor lysis without significant effect on platelet count in three patients 19. Fifty-six previously treated patients were enrolled in a phase-I study of ABT-199 57 producing a 21% CR rate with few grade 3/4 adverse events. Interestingly responses were independent of the presence of 17p− and of fludarabine-refractory disease. Consistent with these results a greater that 87.5% ORR was reported in relapsed/refractory CLL with 17p− and/or TP53 mutation 58.

#### Lenalidomide

Treatment interfering with the interactions of CLL lymphocytes in the microenvironment and the immune system using lenalidomide is under investigation 59,60.

Shanafelt and coworkers 61 reported on a trial of pentostatine, cyclophosphamide, and rituximab as induction regimen followed by lenalidomide consolidation in untreated CLL, showing improvement in the quality of response in 24%.

The association of lenalidomide with rituximab was effective in relapsed/refractory CLL (Table 2), including the 17p− subset, where a 53% ORR was observed 62. Preliminary data showed that this combination was effective in untreated CLL 63.

## 11q−

11q deletion occurs in 10–15% of the cases and involves *ATM*, a principal DNA damage response gene 64–66.

11q− was associated with an inferior prognosis 34,67, however, the combination of fludarabine and alkylating agents improved the outcome in this cytogenetic subset of CLL 68. A 40% CR rate in previously untreated 11q− patients was reported by using bendamustine and rituximab 69 and evidence was provided that the combination of purine analogs with cyclophosphamide and rituximab may overcome the negative prognostic impact of this chromosome deletion 70. An analysis of prognostic factors in the CLL8 trial did not identify 11q− as predictor of a shorter PFS in multivariable analysis 71.

In the relapsed/refractory setting a 92% and 57% ORR were achieved by the combination bendamustine and rituximab 72 and by flavopiridol as single agent, respectively 42.

BTK inhibitor ibrutinib proved effective in this cytogenetic subset, however it is worth noting that, possibly due to clonal evolution, those patients with 11q− or 17p− may progress under ibrutinib more frequently than patients without 11q−/17p− 9. Likewise treatment by idelalisib and ABT-199 proved effective irrespective of the presence of 11q− 53,58.

## SF3B1 and NOTCH1 Mutations

Lesions of *SF3B1*, *NOTCH1*, and *BIRC3* were clearly associated with relapsed/refractory disease 17,73.

The significance of *SF3B1* and *NOTCH1* mutations were studied in 494 patients enrolled in the UK LRFCLL4 trial, randomizing patients to receive chlorambucil, fludarabine, or fludarabine and cyclophosphamide 36. While no difference in terms of ORR was noted in each trial arm for these lesions, *NOTCH1* and *SF3B1* mutations were associated with shorter OS and *SF3B1* gene was associated with reduced PFS in FC-treated patients. Likewise, *SF3B1* mutations showed independent negative prognostic value for PFS in patients receiving first-line FC and FCR treatment in the CLL8 trial 74. Interestingly, *NOTCH1* mutations appeared to identify a subset of CLL patients that did not benefit from the addition of rituximab to FC 74. In line with these findings the adjunct of the anti-CD20 antibody ofatumumab to chlorambucil prolonged significantly PFS in a phase III trial, but this benefit was not observed in those patients with *NOTCH1* mutations 75. In another analysis, 18 *NOTCH1*-mutated patients showed an inferior CR rate under fludarabine associated with alemtuzumab or cyclophosphamide as compared with patients without this aberration 76.

To the contrary, idelalisib, alone in combination with rituximab or chemoimmunotherpy combinations proved equally effective irrespective of the presence of *NOTCH1* mutations in a study including 232 patients 53.

## Conclusion

Modern CLL treatment is a remarkable example of how biologic studies and clinical expertise may converge, providing a rationale basis for the development of effective treatments, resulting in a significant improvement of the number and quality of responses, quality of life, and survival 77 in the majority of age groups, including the elderly population 20,26.

In genetically defined high-risk disease effective first-line chemoimmunotherapy combinations improved the quality and duration of response in 11q− and 17p-/*TP53* mutated cases; allogeneic bone marrow transplantation overcame the unfavorable prognostic significance of 11q−, 17p−/*TP53* mutations, *NOTCH1*, *SF3B1* mutations and very effective compounds targeting the BCR signaling or BCL2, provided excellent and durable results in the setting of chemorefractory disease and in untreated patients 46.

The cost of hemopoietic neoplasms is an issue in high-income countries 78 and the development on novel treatment in CLL is likely to become soon a real challenge for the national health systems 79.

However, it is worth noting that the pharmacoeconomic analysis performed by the National Institute of Health and Clinical Excellence in the United Kingdom recognized that FCR, a regimen improving survival in a direct comparison with the best chemotherapy combination was a cost-effective use of NHS resources. The predicted efficacy of very potent, targeted, and nonchemotherapeutic drugs in CLL along with the development of sensitive predictors of response offer a unique opportunity to intensify coordinated research programmes aimed at providing compelling evidence of the positive cost/efficacy ratio of these novel agents.
